# Medical Students’ Perspectives on Family Planning and Impact on Specialty Choice

**DOI:** 10.1001/jamasurg.2023.6392

**Published:** 2023-12-13

**Authors:** Ebernella Shirin Dason, Madalina Maxim, Dionne Gesink, Michelle Yee, Crystal Chan, Nancy N. Baxter, Heather Shapiro, Andrea N. Simpson

**Affiliations:** 1 Division of Reproductive Endocrinology and Infertility, Department of Obstetrics & Gynaecology, Mount Sinai Hospital, University of Toronto, Toronto, Ontario, Canada; 2 Temerty Faculty of Medicine, University of Toronto, Toronto, Ontario, Canada; 3 Department of Obstetrics and Gynecology, McGill University, Montréal, Québec, Canada; 4 Dalla Lana School of Public Health, University of Toronto, Toronto, Ontario, Canada; 5 Department of Emergency Medicine, Mount Sinai Hospital, University of Toronto, Toronto, Ontario, Canada; 6 Li Ka Shing Knowledge Institute, St Michael’s Hospital/Unity Health Toronto, Toronto, Ontario, Canada; 7 Melbourne School of Population and Global Health, University of Melbourne, Melbourne, Victoria, Australia; 8 Department of Obstetrics and Gynaecology, St Michael’s Hospital/Unity Health Toronto, University of Toronto, Toronto, Ontario, Canada

## Abstract

**Importance:**

Physicians are known to delay childbearing compared with nonphysicians and to experience higher rates of age-related pregnancy complications. Delay of childbearing is more pronounced in surgical specialties, and family planning and building goals may influence specialty choice.

**Objective:**

To assess medical students’ perspectives on the development of family planning goals and the timing of family building within a medical career to elucidate how these perceptions impact their choice of specialty.

**Design, Setting, and Participants:**

This qualitative study included fourth-year medical students at the University of Toronto Temerty Faculty of Medicine, Toronto, Ontario, Canada, and was conducted between May and August 2021. Participants were purposively sampled to maximize diversity of gender and specialty choice. Interviews were conducted via videoconferencing software that were recorded, transcribed verbatim, and verified for accuracy. Thematic analysis was completed independently by 2 researchers and consensus on final themes was reached through discussion among study investigators. Data were analyzed between September and December 2021.

**Main Outcomes and Measures:**

Participants were asked to share their perceptions of personal family planning goals, support currently in place, family planning education in medicine and factors contributing to their choice of specialty and program. Thematic analysis was completed.

**Results:**

A total of 34 fourth-year medical students (median [range] age, 26 [24-33] years; 23 females [67.6%]) were interviewed. Four main themes were identified: (1) there is no ideal time to family build in a medical career, (2) family planning is a taboo topic, (3) surgical specialties offer less support for family building, and (4) residents who have children are perceived to place a burden on their colleagues. Medical students considered their family planning while deliberating among specialty choices and their experiences were highly influential in shaping their specialty selection.

**Conclusions and Relevance:**

Results of this qualitative study suggest that medical students perceive that family building during training may have unfavorable implications for team dynamics and relationships with colleagues, and these perceptions may affect specialty choice and family planning goals. Integration of family planning discussions and support for family building into medical curricula is needed along with efforts to improve culture by supporting team dynamics and workload when students take parental leave.

## Key Points

**Question:**

What are medical students’ perspectives about family planning within a medical career and do these perspectives affect their choice of specialty?

**Findings:**

This qualitative study of 34 fourth-year medical students identified 4 major themes from interviews about perspectives on family planning: (1) there is no ideal time to family build in a medical career, (2) family planning is a taboo topic, (3) surgical specialties offer less support for family building, and (4) residents who have children are perceived to place a burden on their colleagues.

**Meaning:**

Findings of this study suggest that the medical system discourages medical students from achieving their family planning goals within training, which may affect their specialty choice.

## Teaser

This qualitative study explores perspectives about family planning within a medical career among fourth-year medical students and their implications for specialty choice.

## Introduction

Physicians undergo rigorous training spanning 10 to 15 years, most of which occurs during their optimal reproductive window (age, 20-35 years).^1^ Up to 84% of physicians find the timing of childbearing challenging,^2^ and many physicians choose to delay childbearing until completion of training.^3,4^ Delayed childbearing may have substantial consequences, including infertility and pregnancy complications.^5,6^ Advanced reproductive age is associated with an increase in adverse outcomes, including preterm birth, preeclampsia, miscarriage, stillbirth, genetic disorders, and severe maternal morbidity and mortality.^5,7,8,9,10^ A prior study indicated high rates of regret among female physicians who delayed reproduction to complete their training, many of whom wished they had attempted conception sooner or gone into a different specialty.^5^

The decision to delay childbearing appears to start early, with only 2% of physicians having children during medical school.^3^ Physicians specializing in family medicine or obstetrics and gynecology (OB/GYN) were more likely to have a child during residency than those who pursued surgical specialties, such as general surgery or urology.^4^ Physicians often experience workplace stigma associated with pregnancy, lactation, and childcare, and they perceive a lack of support from program directors and peers.^11,12^ Residents who face these challenges are more likely to experience greater professional dissatisfaction, burnout (emotional, physical, or mental exhaustion caused by prolonged stress), and decreased wellness.^11,13^ These difficulties are especially pronounced in surgical specialties.^14,15^

Our objective was to conduct semistructured interviews with a diverse sample of fourth-year medical students to understand how students developed their family planning goals and the timing of family building in the context of specialty choice. This knowledge may be used to inform strategies to ensure medical trainees are supported in having children at any career stage regardless of specialty choice.

## Methods

For this qualitative study, we used basic qualitative description, an approach that is widely used in health services research, to gather explicit accounts of experiences and insights on solutions to problems.^16,17,18,19^ To optimize rigor, we followed the Consolidated Criteria for Reporting Qualitative Research (COREQ) reporting guideline.^20^ This study was approved by the University of Toronto Research Ethics Board. Participants provided verbal informed consent, including consent to publish deidentified interview data.

### Sample and Recruitment

We used network sampling to recruit a diverse group of final-year medical students from the University of Toronto Temerty Faculty of Medicine, Toronto, Ontario, Canada, who had matched to residency programs. The study was advertised via email sent to all students from the university’s listserv and through social media pages. Snowball sampling, in which existing participants recommended additional participants, was used to ensure specialty and gender-diverse representation. Interested students were instructed to contact the research team and were asked to answer questions regarding their age, gender, specialty, and program choice. We aimed to involve a minimum of 15 participants; the final sample size was based on informational saturation assessed through theme discussion among the authors. Interviews were conducted between May and August 2021. Participants received a $25 gift certificate.

### Data Collection

The semistructured interview guide was developed through research team discussion, iterative review, and modifications after the first 3 interviews (eMethods in Supplement 1). Interviews were conducted by a senior resident in OB/GYN (E.S.D.) or a second-year medical student (M.M.) via Zoom (Zoom Video Communications, Inc). Self-reported demographic data included age, gender (male or female), sexual orientation and gender identity (heterosexual or lesbian, gay, bisexual, transgender, queer, or questioning), race and ethnicity (East Asian, Middle Eastern, South Asian, White, or other [including Ashkenazi Jewish, Chaldean, Latina, or multiracial]), and choice of specialty. Race and ethnicity were included to ensure a diverse sampling of people, as ethnicity may influence family planning goals. Questions assessed the participant’s knowledge of family planning support in residency, family planning education, attitudes around family planning, their personal family planning goals, and factors they considered in selecting their specialty and residency program. Interviews lasted 30 to 60 minutes and were recorded, transcribed verbatim via Zoom, and verified for accuracy by one of us (M.M.).

### Data Analysis

We used descriptive thematic analysis, meaning themes were not determined beforehand but rather emerged from the data.^21^ Thematic analysis uses open coding, constant comparison, and axial coding to identify common and divergent themes (with particular focus on male vs female participants) that characterized the entire data set in relation to major categories, such as perceptions and discussions around family planning and how family planning may relate to specialty choice.^21,22,23^ Thematic analysis and coding were done by 2 of us (E.S.D. and M.M.) independently using NVivo, version 12 (Lumivero). The research team met to compare common themes and coding strategies. The entire team reviewed the data and interpreted findings. Data were analyzed between September and December 2021.

## Results

Fifty-one students expressed interest in this study. We interviewed 34 fourth-year medical students (median [range] age, 26 [24-33] years; 23 females [67.6%] and 11 males [32.4%]) pursuing a variety of specialties: 29.4% from OB/GYN and surgical specialties, 47.1% from medical specialties, and 23.5% from family medicine (Table 1).

**Table 1.** Demographic Characteristics of the Study Sample of Fourth-Year Medical Students

Characteristic	No. (%) (N = 34)
Gender	
Male	11 (32.4)
Female	23 (67.6)
Age, median (range), y	26 (24-33)
Sexual orientation and gender identity^a^	
Heterosexual	30 (88.2)
LGBTQ	3 (8.8)
Not disclosed	1 (2.9)
Race and ethnicity^a^	
East Asian	10 (29.4)
Middle Eastern	2 (5.9)
South Asian	8 (23.5)
White	10 (29.4)
Other^b^	4 (11.7)
Specialty^a^	
Family medicine	8 (23.5)
Internal medicine	4 (11.8)
Medicine, nonprocedural^c^	7 (20.6)
Medicine, procedural^d^	5 (14.7)
Obstetrics/gynecology	6 (17.6)
Surgery^e^	4 (11.8)

Abbreviation: LGBTQ, lesbian, gay, bisexual, transgender, queer, and questioning.

^a^ Percentages may not add to 100 because of rounding.

^b^ Other race and ethnicity includes Ashkenazi Jewish, Chaldean, Latina, or multiracial.

^c^ Psychiatry, pediatrics, and physical medicine and rehabilitation.

^d^ Dermatology, anesthesia, and emergency medicine.

^e^ General surgery, vascular surgery, and otolaryngology–head and neck surgery.

We found a central theme: medical students perceived that family planning or building was not well supported within a medical career, especially during training. Four themes supported this conclusion: (1) there is no ideal time to family build in a medical career, (2) family planning is a taboo topic, (3) surgical specialties offer less support for family building, and (4) residents who have children are perceived to place a burden on their colleagues. Two-thirds of participants (22 of 32) considered their family planning goals during specialty selection.

### Theme 1: There Is No Ideal Time to Family Build in a Medical Career

Participants felt their choice to pursue medicine represented a delay in family planning compared with their nonphysician peers, and thus a career in medicine changed their ideal family plan. They compared the ability to family build during medical training and after medical training. Factors they considered during family planning included the availability of parental leave, return to work as a parent, work-life balance, financial stability, the structure of medical training, the physical demands of pregnancy, and childcare (Table 2).

**Table 2.** There Is No Ideal Time to Family Build in a Medical Career

Subtheme	Supporting quotes
Structured academic commitment, stress to perform well	“Medical school itself is too demanding on our time and I don’t find that the school’s terribly flexible in sort of adapting to people’s lives and schedules. And frankly I just couldn’t take on that extra burden or responsibility of having a kid on top of all of the things expected from us in medical school.” (Female participant)
Financial burden, financial stability, affordability of children	“I didn’t have any income and I’m sure you know as well, without an income, it does feel a little bit uncomfortable to have kids because kids, they’re not cheap, they’re very expensive.” (Female participant)
Physical implications of pregnancy for skills development	“For females that is a consideration, like, I’m going to be out of this environment for weeks, for months. That really deters them and I’m going to be, like, sick operating with nausea before I leave, I’ll just not be performing my best.” (Female participant)
Difficulty balancing work and parenting commitments	“People mentioning how difficult it is to be a mom working in medicine with demanding hours especially in the surgical field and have the competing, competing interests of, like, family planning and raising children. And also, just like, I’ve heard a lot of female physicians discuss like, having to miss important events of their children’s lives because their schedule isn’t always their own.” (Female participant)
Having children adds to overall exhaustion	“[The attitude] is mostly that you know, it’s just manageable but it’s very exhausting. And I think most of the residents that I interacted with who already had children just talked about it being a lot to balance.” (Female participant)
	“Just because, you know, all residents are like, they’re not not busy, like they’re quite busy people. And with that being said, like, even people who are not in medicine kind of have that same, you know, tone of, you know, exhaustion about it too. And maybe this is just like, like a general, like cultural, thing, and you know, in our society or whatever, or you know, the professional class of folks. But like, I don’t, like no one’s really like... I don’t know, no one’s really like super like hyped, or like excited for like, family planning. It just kind of seems like, you know, something that you’re trying to fit into your life but it’s difficult to fit into your life.” (Male participant)

Paid parental leave availability during training was viewed favorably by participants, in contrast to the lack of income during parental leave as a practicing physician. Many participants viewed parental leave during training as difficult due to the rigidity of training programs, possible loss of skills, and the need to extend training pathways. Students felt there was little support on return to work in medical training in terms of lactation, childcare, and work-life balance. Several students felt that the balance between clinical and parenting duties continued to represent a challenge as a practicing physician. Participants commented that while practicing physicians may have more flexibility in their schedule to allow for a graduated return to work, the medical education culture discourages time away from work for personal commitments, especially as an early-career physician.

Medical training also represented a financial burden. Many students described having debt, and having a child represented an additional financial burden that they did not feel they could carry while in training. Students shared that they were not aware of what their future level of income may be and when they may achieve financial stability.

Students emphasized that medical school is an intensely structured academic commitment without schedule flexibility that is accompanied by the stress of performing well. Students commented that clinical and academic performance within medical school was linked to becoming a desirable candidate for residency program. Some students felt they also had to engage in extracurricular commitments (eg, examinations, research), which leaves little time for parenting. Students felt that they needed to perform at higher standards of professionalism to be considered as candidates for a particular residency program. They shared that residents may not have to perform to those standards since the pressures of competing for a position are no longer present. Students viewed requesting time away from clinical duties for medical appointments, parental leave, and parenting responsibilities and the physical restrictions of pregnancy as sources of stress, primarily due to the fear of undesirable judgment by preceptors and peers.

Students recognized that pregnancy also represented a physical burden (ie, nausea, physical restrictions) that may have implications for their ability to perform well in their training role and gain appropriate surgical skills. People who are pregnant require frequent medical appointments, which may be difficult to arrange within a rigid training program. Therefore, pregnancy also represented time away from training for many students, in addition to parental leave.

Lastly, students highlighted that childcare was a substantial source of stress to balance with a medical career. Students commented on the difficulty in arranging childcare due to unpredictable schedules. In training, students considered programs geographically closer to family support, especially when considering family building. Some students commented specifically on choosing a nonphysician partner to allow for more flexibility with family building.

### Theme 2: Family Planning Is a Taboo Topic

Participants largely felt that the current culture within medicine discouraged the open discussion of family planning. The onus to find information about how to family plan and family build was primarily placed on individuals. Many participants were unsure how to find this information. Thus, perceptions of family planning and the compatibility of family building within a medical career were developed through informal discussion initiated by personal mentors or raised during clerkship experiences with supervising faculty and residents (Table 3).

**Table 3.** Family Planning Is a Taboo Topic

Subtheme	Supporting quotes
Seeking out family planning information informally	“I sneakily slide it in during all my electives.” (Male participant)
	“So many people kind of say things like, ‘Oh, you want kids, that’s difficult to do.’ But like at the same time, almost everyone I worked with had a family, so no one really talks about, like, supports they offer in family planning.” (Female participant)
Encouragement for family planning	“The attitude always, it always seemed overwhelmingly positive and nobody really, nobody from what I remember ever seemed to say that it was a detriment to their career, or it was a problem, or a cautionary tale.” (Male participant)
Discouragement for family planning	“I think that’s like a lot of women who are in this situation have a negative attitude, because a lot of the duty falls to them just in our society and so they’re not necessarily the happiest with that. Some women have been, like, more about a warning, you know, just so you know this is kind of in store for you.” (Female participant)
Concerns about reputation	“I think there is also a little bit of a perception that maybe it’s not the smartest idea to ask about that in interviews. Because, you know, you hear whispers, and this is obviously not allowed, but you do hear whispers of programs preferring other applicants who maybe are not planning to take parental leave. It’s something that we’re not really advocating for ourselves to program leadership for fear of repercussion.” (Female participant)
	“One of my friends had a small group session with a surgeon who was once on the board of an admission committee for a surgical residency program. They were just chatting and they asked him, ‘What’s something you wish you knew about an applicant but don’t ask?’ And he said, very explicitly, that he wishes he could ask if the applicant was planning to take parental leave, because he said once residents take parental leave that they very infrequently finish a surgical residency. They always invariably switch to something ‘easier’ and honestly it was really very inappropriate, but I think it does reveal the sentiment and this fear that we all had that there would be this kind of bias that programs would hold against these plans. But that was just confirmed in that interaction and I’m not surprised that was the case.” (Female participant)
	“I think that we were concerned that it’s lip service for the faculty to say that [they support family planning], then [the institution gets] accredited and doesn’t get complaints, vs in reality the culture hasn’t actually changed to be one that was accepting and understanding. Which sounds very cynical, but I think that is how we thought of it and we wanted to protect ourselves in that regard.” (Male participant)
Age-related fertility decline causes anxiety	“My partner and I were shocked by this—I didn’t realize what the classic reproductive window was until I got into medical school. I always thought I had a lot more time and then I remember in first year we saw some graph about, like, what happens after 35 in terms of, like, pregnancy-related complications, difficulty conceiving, and I remember, like, all of my friends and I started thinking, like, oh my God, like, should we freeze our eggs? How are we going to have, like, how are we going to get it done in time?” (Female participant)
Unfair burden on women, lesser burden on men	“I think, as a, one the woman who’s actually having the baby, you can’t just be like, ‘no, I can’t be, like, in labor right now,’ it’s not something in your control. You can’t cancel your prenatal appointments, you can’t not breastfeed when, you know, every 2 hours, when you need to. But I also think there’s this societal expectation that if you’re a mom and you’re not spending enough time with your kids, you’re a bad mum, but if you’re a dad and you’re not spending time with your kids, you’re a hard worker and you’re, you know, working toward something to support your family.” (Female participant)
Need for recognition of male role in parenting	“I think the impact that pregnancy has on men’s lives, especially in the beginning of the child’s life, is much less than the impact it has on the woman’s life, just because of, like, biological needs of the child. So, I think from that aspect, it definitely doesn’t get brought up as much. I saw during my [general surgery rotation], though, because my senior resident did have a kid and he wanted to take more time off and he only got, I think, 2 weeks. Because that’s all they would allow him and he said, like, ‘I want to take more, I wish I could take a month, but then it kind of like pushes residency’ and it was general surgery, there wasn’t much kind of flexibility.” (Male participant)
Desire for explicit information about family planning	“Yeah, I think I would have liked to see [the need for information about family planning] being acknowledged more openly to people. I think that if more people knew, okay, you can have a kid and you won’t get kicked out of medical school—I’m being dramatic—but, like, you can have a kid and you can complete medical school still in a reasonable time frame and still match to whatever specialty you want. I wish that was made more public so that people, particularly women, who are maybe a little bit older in the class, could seriously consider that. I have literally no idea if [medical school will] offer that family support.” (Female participant)
	“I would like there to be a sort of a more open conversation about these things. That’s something that medicine is missing in general, like just these more fulsome conversations about, like, not medicine and not career necessarily related things. Just like sort of personal life things and like, how folks that have gone through this, like, family planning. I think having even just, like, mentors or folks that are open to be, like, reached out to would be, like, really helpful. Because again, I really put a lot of value in those sorts of, like, informal relationships and I think having those conversations is really nice. I think that would be really helpful because it puts the onus on the program to start a conversation and not necessarily on the resident who’s already sort of in a lower position of power and is already trying to navigate, like I said before, like a fairly conservative work culture. So, I think it’s a place to start, like obviously I don’t think it’s enough but it’s already, like, more than programs are already doing.” (Male participant)
	I think, maybe just talking about it a bit more would be a great first step. I think there is a little bit of like this taboo-ness and hush-hush around the topic. And it’s probably for very complicated reasons, but I think, you know, it’s definitely something that I, and a lot of my female classmates, have thought about throughout [medical] school but it’s not really something that was ever talked about openly, aside from the smaller circles. So, I think removing that stigma that we’re starting to talk about, it would be a positive first step.” (Female participant)
	“I think maybe just more from, like, a planning perspective, like hearing other perspectives from physicians, like how they factored into their career decision-making, or perhaps training decisions, how they were able to juggle those commitments between, like, between training and having a family, I think is useful. Just I think for me, hearing from other people’s experiences and how, when was the right time for them and how they factored that into their decision-making is probably most useful.” (Male participant)

Students found that mentors’ attitudes, whether encouraging or discouraging toward family planning, highly influenced their personal views. Female participants valued cisgender role models who were open to discussing parenting in medicine. Male participants wished parenting in medicine was discussed with them at the same level that it was discussed with female students.

Students described that while the onus is placed on them to seek information rather than the information being openly provided, they felt they had to ask questions discreetly because they were worried about how a desire to have children during training would be perceived. Through informal conversations, participants felt that their professional reputations may be at risk should they discuss family planning; this was true for both male and female participants.

Medical students also shared how age-related fertility decline is communicated with them. This decline is largely not acknowledged on a personal level, but rather is simply one element of the vast medical curriculum. For many students, this topic created anxiety as it was introduced medically but not explored personally. The overwhelming majority of participants felt that family planning in medicine should be acknowledged explicitly.

### Theme 3: Surgical Specialties Offer Less Support for Family Building

All participants shared that aside from personal anecdotes of the burden or joy of having children, medical school and residency programs were not explicit about support provided for trainees engaging in family building. Thus, students formed their opinions about the ability to family plan within specialties based on their personal experiences and informal mentorship. Even 1 conversation about the compatibility of family building with a specific specialty could be highly influential for a student, which contributed to persistent stereotypes. Surgery, in particular, was seen as a specialty in which family planning was generally discouraged both for males and females. Students who were motivated to pursue family building were more likely to choose a specialty and program that they perceived as more supportive of their goals. Students who were less motivated to pursue family building still balanced thoughts about whether their program choice would be supportive or not, and this consideration had implications for their family planning (Table 4).

**Table 4.** Surgical Specialties Offer Less Support for Family Building

Subtheme	Supporting quotes
Specialty stereotypes, single influential conversation or experience	“For internal medicine, when I spoke with, like, female residents and I asked, are you, like, able to take time off, example [maternity] leave during my residency, is that a thing? And they said nope, I have not heard of a single female resident who took off time for [maternity] leave during residency so that was a factor to me.” (Female participant)
	“I remember talking to a [medical] student about their surgical rotation and they said that their surgical staff said, don’t go into surgery if you want to have a family. And this is obviously just one staff, but you know, hearing things from people who are in that position telling you, like, ‘oh don’t do this if you, like, care about having a family.’” (Female participant)
	“And I know that obstetrics and gynecology is an extremely demanding specialty, and the hours might not be as… or suited to having a family, compared to a specialty like family medicine or pediatrics.” (Male participant)
	“I have a very distinct image that I won’t forget of 2 pregnant women who were operating. One was the staff and one was the resident, and both were about 30 something weeks’ pregnant. And I was worried for them, because this is a 6- to 7-hour surgery, and they did not eat. So I was very worried and with a nurse, we had to get juice for them in the middle, because they did not look well but they pushed through. So it’s doable for those who are very determined, but I don’t think for me, I would want to do that just because physically I’m not sure if I’m going to be able to handle it—those were some incredible women. But also having to take that time off and go home, raise that child, and come back to the [operating room] while still raising, like, a toddler, it just makes me wonder how they get that balance.” (Female participant)
Surgical career is not conducive to family planning	“I think in surgical specialties, you dedicate your life to becoming a surgeon, and so that’s my understanding and that’s okay with me.” (Female participant)
	“There are certain, like, more male-dominated fields, like, talking to neurosurgeons where, it’s you know, the discussion of fertility and things like [maternity] leave are, like, a little scarier. And I think, you know, women in medicine do sometimes think about being a burden … to their program but at the same time also will I be a good parent, like, what can I manage, like, this work-life balance between the home.” (Female participant)
	“There are some surgeons who completely would disagree that their specialty doesn’t have room for a balanced lifestyle. But there are others who said, like, if you do things like surgery, like, it kind of becomes your whole life. Whereas nobody in psychiatry or family medicine ever told me, like, you know, psychiatry will take over your life or nobody ever said, like, you have to eat, sleep, and breathe family medicine for the rest of your life if you do family [medicine].” (Male participant)
Hidden curriculum regarding required activities for a future career	“Doing a surgical specialty, particularly in an academic center, there’s sort of the hidden curriculum, even as a resident and a staff, that you need to take on other duties, you need to be involved in research, you need to be involved in medical education, need to be involved in sort of administrative duties, and those things really add on to your workload and add on to the, all of these expectations you have on your plate from a work perspective and that’s something I’ve seen with my own eyes shadowing, doing electives, doing rotations and I’ve heard from staff and I’ve heard from residents. I’ve seen so many [obstetrics] residents, like, break down and cry on their shift from being just stressed out and on sort of their breaking point of what they can handle.” (Female participant)
Influence of a single mentor, impression of having children in medicine	“This is something actually witnessed on my shadowing experiences in second year, one of the residents actually was having twins at the time. I’d asked her what her experiences were like going throughout residency, you know, going through and dealing with the twin pregnancy and she had nothing but positive things to say about the program being supportive of her, allowing her, you know, kind of as much time off as she needed. And so from kind of my experiences I’ve heard great things and kind of anecdotally I’ve seen that as well.” (Male participant)

Students shared that they had met residents and staff physicians who were pregnant or had children but had not necessarily been involved in explicit conversations about their experiences. Students would then interpret the experience of being a parent in medicine based on their impressions about their supervisor’s life. Participants’ impressions of surgeons who were pregnant or had children were generally more unfavorable, and they discussed concerns around the ability to balance a surgical career during pregnancy or with young children.

### Theme 4: Residents Who Have Children Are Perceived to Place a Burden on Their Colleagues

Participants perceived that trainees who have children place a burden on the medical system and contribute to the burnout of their peers. This perception perpetuated the fear of concerns about one’s reputation because students who take parental leave are more likely to be viewed unfavorably. Students shared that having children is considered a personal choice that makes one less of a team player. The general messaging that students heard is that having children during residency disrupts the structure of the training program and peers are not, and perhaps should not, be supportive. Importantly, these experiences led students to seek out larger programs due to the perception that the work was better distributed and thus parental leaves would be less likely to harm their peers. Students also sought out specific specialties that had lighter work-hour requirements and less interdependence between residents (Table 5).

**Table 5.** Residents Who Have Children Are Perceived to Place a Burden on Their Colleagues

Subtheme	Supporting quotes
Having a child is a burden on or unfair to others	“Even now, I do think it’s unfair, like, if you are, for example, 3 people in a program. And there’s, like, 3 females, and 1 of them decide to have a kid, I think it’s unfair to ask the other team to pick up the call slot if that person has, like, a childcare emergency for, like, I think no one else should be getting punished for someone else’s life decisions. Because I think that, like, one of the problems in surgical residencies is that it’s going to cause burnout in the other residents if they have to be on call every 1 in 2 days, because others have issues because they’re having a kid because it’s, like, that was their personal decision.” (Female participant)
Size of program influences burden on colleagues	“I think so. I think, maybe less so for me, since my kind of future residency program is, it’s very big and I think I’ve also heard of a couple of people who do take parental leave during internal medicine residency. So, I think that barrier for me is maybe a little bit less since I know people who’ve done it before and it’s kind of big enough that you know you don’t feel like, okay, well, you’re 1 of 3 residents and now it’s down to 1 in 2 and then there’s a problem. But I can definitely see how that would impact others in smaller programs for sure.” (Female participant)

## Discussion

Findings of this qualitative study suggest there is a hidden curriculum in medicine that discourages medical students from family building during medical training and affects students’ family planning overall. Students begin their medical career with the concept that a medical career is not congruent with their ideal family plan. They then develop an understanding through the hidden curriculum that there is little opportunity to balance family and a career in medicine. There is a substantial onus placed on the individual to set goals and be resilient to achieve parenthood in a medical career. This feeling is especially pronounced in surgical specialties. Family planning within a medical career was important to both male and female students, and males described fewer opportunities to discuss family planning than females had.

Previous literature has highlighted that physicians regret delaying childbearing and experience higher rates of infertility and miscarriage, likely associated with age-related fertility decline.^5,13^ Trainees face substantial difficulties with having children during residency.^11,13,24,25^ Findings of the present study suggest that the general discouragement toward family planning begins in medical school. In Canada, there are supports for generous paid parental leave in training, yet participants in our study continued to receive the message that taking parental leave is not necessarily supported by the wider medical community.^26^ It is likely that trainees in jurisdictions with no paid parental leave during residency experience additional challenges. Difficulties with family planning expressed by participants of the present study, including a busy work schedule and a desire not to extend training, are messages circulating through the hidden curriculum from supervisors and peers.^24^ Medical students were concerned about returning to work with young children due to long training hours, workload, extracurricular expectations, financial instability, and childcare. Strategies beyond paid parental leave are needed to truly support parenthood within medicine.^11,27^

Changing medical culture and improving support for all physicians to achieve their family goals should start with explicitly addressing the discourse with trainees. Currently, conversations about family planning are led informally by peers and supervisors, with their opinions and experiences affecting the perceptions of students. Medical students are hesitant to ask questions about family planning due to the stigma surrounding the topic and concerns about their reputation in the context of competing for residency positions. Students expressed the need for formal curricula, policies, and a culture shift to support family building within a medical career. We believe that information about engaging in family planning should be widely available to students, and formal opportunities to discuss family planning in the context of a medical career should be available through career counseling.

Another opportunity to improve culture is to address the perception that physicians with children are burdensome to other team members. Findings of the present study support those of previous studies,^11,24^ with residents highlighting the hidden curriculum in the ongoing passage of these values to medical students. Workplace climate (autonomy, workload, collegiality) and team dynamics have been shown to affect job satisfaction, resident burnout, and ultimately patient care.^28,29,30^ While there is a heavy focus within residency programs to build individual resilience, systemic factors largely influence physician burnout.^30^ In the current constrained medical system, there is an overreliance on trainees, such that a single absence overburdens the remaining team.^11^ Taking pregnancy or parental leave is therefore perceived as a disruption to the team, adding workload to peers.^11^ Interventions to mitigate the consequences of a trainee taking parental leave may therefore improve culture and reduce burnout for all trainees involved. Such interventions may include auditing the number of residents on parental leave and ensuring adequate workforce availability despite leaves, reducing workload for residents through off-loading tasks to other health care team members (ie, physician assistants), considering extra compensation for rescheduled call shift coverage, work-hour limitations that take childcare into account, and improving interpersonal dynamics among resident teams.^30^

Medical students believed that surgery was the specialty choice that was least conducive to family building. They perceived less willingness to openly discuss balancing a surgical career with family planning goals, and received unfavorable messaging about how the desire to have children affected reputation and thus candidacy for a career in surgery. Female students were specifically fearful of how pregnancy affects surgical training and skills attrition during parental leave. These experiences are highly influential in shaping a students’ view of a surgical specialty and the support of family building. Overall, open discussions, leadership commitment to support individual autonomy, appropriate distribution of workload, and an adequate workforce are important goals for addressing the surgical workplace culture and thus changing implicit messaging conveyed to students. It is critical that change occurs across the entire medical culture, particularly in surgical disciplines, and not just for trainees. It is possible that some institutions have already made these internal efforts, in which case, further efforts should be directed toward explicit messaging about family building supports in training and how to balance parenting duties with clinical duties. Such efforts may encourage more students to consider surgical specialties.

### Strengths and Limitations

Strengths of this study include its broad sampling of students at the largest medical school in Canada, with a specific aim to be gender inclusive. Additionally, a rigorous qualitative methodological approach was applied.

The study also has limitations. Although thematic saturation was achieved, it is possible that we did not capture all possible viewpoints; for example, we did not extend the study to include other medical schools. However, the University of Toronto Temerty Faculty of Medicine represents a large, diverse training center and is likely representative of medical culture in other jurisdictions. We did not capture aspects of family building during other career stages; additional research is needed in this area. Exploration of family planning during training in settings outside of Canada is needed, as other countries may differ in the allowable length of parental leave, which may affect the timing of family building. Next steps include assessing current family planning and family building support among residents and faculty, assessing specific interventions to improve family planning support for trainees and faculty, and developing a framework for best practices in trainee support for family building.

## Conclusions

This qualitative study found that there is a hidden curriculum in medical school that actively discourages family building during training, particularly in surgical specialties. Family planning remains a taboo discussion topic for medical students, who are concerned that such discussions will reflect unfavorably on their reputation, as residents who have children are perceived to disrupt team dynamics. These perceptions may affect specialty choice and family planning goals among medical students. We propose 2 initial interventions to address the need for family building discussions during medical training. First, open discussions about family planning within a medical career and support for family building should be incorporated into medical school curricula and continue into residency within all specialties. Second, efforts to improve culture through innovative interventions to support team dynamics and workload when a trainee is on parental leave are needed.

## Notes

Supplement 1. **eMethods.** Interview Questions

Supplement 2. Data Sharing Statement
