# Supplementary material for: Medical Students’ Perspectives on Family Planning and Impact on Specialty Choice
Source: JAMA Surg. 2023 Dec 13;159(2):170–8. doi: 10.1001/jamasurg.2023.6392 (PMC10719828; doi:10.1001/jamasurg.2023.6392)
Supplement: Supplement 1. — eMethods. Interview Questions [file jamasurg-e236392-s001.pdf]

Supplemental Online Content

Dason ES, Maxim M, Gesink D, et al. Medical students' perspectives on family planning and impact on specialty choice: a qualitative study. *JAMA Surg.* Published online December 13, 2023. doi:10.1001/jamasurg.2023.6392

eMethods. Interview Questions

This supplemental material has been provided by the authors to give readers additional information about their work.

©2023 Dason SE et al. *JAMA Surgery.* All rights reserved.

eMethods. Interview Questions

Introduction: The following questions explore your personal thoughts on family planning and your knowledge on fertility changes and treatments. Please consider the answers as they pertain to you, not to future patients you may be counselling. You may choose not to answer questions or take a break if you need to. It may be difficult to share your experiences with me.

Residency-specific Questions

1. Which residency programs did you choose to apply to?
2. Tell me how factors such as family planning, lifestyle and work-life balance contributed towards your specialty selection.
3. How did considerations around the hours worked or length of residency play into your decision to select a specialty/program?
    a. Which of these factors (ie. Family planning, lifestyle, work-life balance, hours worked, length of residency) would you say was the most important?
4. Did you consider whether or not your specialty would have family planning supports in place when selecting your residency?
5. How did you seek information about the specialty you applied to?
6. How did you seek information about the programs you applied to?
    a. Where did people develop their viewpoints on how supportive a speciality was on their goals regarding family planning?
7. Did you have any specific mentors that you sought information from?
8. What factors did you consider most important when you were ranking programs?
    a. Did you consider the “culture” of the program?
9. How did your relationship status impact your residency specialty or program decision?
    a. Are you in a relationship? (If so) What does your partner do? (If they are in medicine) Did you couples match?
10. Do you think your residency program that you have matched to would be supportive of residents having children during residency?
    a. Do you know what supports are available to residents having children?
    b. Do you know what supports are available to residents who want to preserve their fertility?
    c. Were the programs you applied to explicit in sharing this information with you?
11. What kind of supports would you like to see in your residency program?
12. How do you imagine that family planning as a staff will look like?

Medical School Specific Questions

1. What does your medical school communicate to students/residents about family planning and having children?
2. What family planning supports are available to students?
    a. Do supports differ by gender?
3. How has medical school impacted your family planning?

©2023 Dason SE et al. *JAMA Surgery.* All rights reserved.

4. (If they have a child) What was your experience having a child/raising a child before or during medical school? Was the school supportive? Did you feel supported by other medical students?
5. What kind of supports would you have liked to see in medical school?
6. (If they have a child) What would you want to say to another medical student who had a child or is planning to start their family?

Fertility-related Questions

1. What do you know about family planning and fertility?
    a. How would you rate your knowledge on family planning and fertility, 1 to 10?
2. What is your understanding of age-related fertility changes?
    a. What are your thoughts about age-related fertility changes in the context of your own life?
3. Was having children in the context of a career in medicine discussed formally during your training? (ie. Lectures, material)
    a. Was it ever informally discussed? Preceptors, medical students, family?
    b. Was this ever discussed? How was this discussed?
    c. What was the attitude of those with who you discussed this?
    d. Did you seek out information/mentorship around these issues during medical school? Before medical school?
    e. What did you learn and how did what you learned impact your perceptions?
4. Would you tell me your what your thoughts are on when you might choose to have a family?
    a. What factors influenced your decision regarding when to have a child?
    b. Did your age, gender, sexuality, or ethnicity have any impact on your goals?
5. What is your understanding of the family building options that are available to you? (i.e. spontaneous conception, IVF, egg-freezing, adoption, surrogacy)
6. Have you considered whether you might personally encounter fertility issues during your training?
7. Have you considered fertility preservation through elective egg freezing for you or your partner or IVF during training?
8. Is there an age at which you would you think eggs should be freezed if you are not ready to have children?
    a. What would motivate you to freeze your eggs?

Conclusion:

Do you have any questions for us? Thank you for the time you've taken to share your thoughts with us.

©2023 Dason SE et al. *JAMA Surgery.* All rights reserved.
