# Supplementary material for: Medical Students’ Perspectives on Family Planning and Impact on Specialty Choice
Source: JAMA Surg. 2023 Dec 13;159(2):170–8. doi: 10.1001/jamasurg.2023.6392 (PMC10719828; doi:10.1001/jamasurg.2023.6392)
Supplement: Supplement 2. — Data Sharing Statement [file jamasurg-e236392-s002.pdf]

Data Sharing Statement

Dason. Medical Students' Perspectives on Family Planning and Impact on Specialty Choice. *JAMA Surg*. Published December 13, 2023. doi:10.1001/jamasurg.2023.6392

Data
Data available: No
